# Supplementary material for: Rapid summer Russian Arctic sea-ice loss enhances the risk of recent Eastern Siberian wildfires
Source: Nat Commun. 2024 Jun 26;15:5399. doi: 10.1038/s41467-024-49677-0 (PMC11208637; doi:10.1038/s41467-024-49677-0)
Supplement: Supplementary file 1 — Supplementary Information [file 41467_2024_49677_MOESM1_ESM.pdf]

SUPPLEMENTARY INFORMATION

Rapid summer Russian Arctic sea-ice loss enhances the risk of recent Eastern Siberian wildfires

Binhe Luo[1)], Dehai Luo[2)], Aiguo Dai[3)], Cunde Xiao[1)], Ian Simmonds[4)], Edward Hanna[5)], James Overland[6)], Jiaqi Shi[2)], Xiaodan Chen[7)], Yao Yao[2)], Wansuo Duan[2)], Yimin Liu[2)], Qiang Zhang[8)], Xiyan Xu[2)], Yina Diao[9)], Zhina Jiang[10)] and Tingting Gong[11)]

(1) State Key Laboratory of Earth Surface Processes and Resource Ecology, Beijing Normal University, Beijing, China
(2) Institute of Atmospheric Physics, Chinese Academy of Science, Beijing, China, 100029 and University of Chinese Academy of Sciences, Beijing, China
(3) Department of Atmospheric and Environmental Sciences, State University of New York, Albany, NY, USA
(4) School of Geography, Earth and Atmospheric Sciences, University of Melbourne, Melbourne, Australia
(5) Department of Geography, School of Life and Environmental Sciences, University of Lincoln, UK
(6) NOAA/Pacific Marine Environmental Laboratory, Seattle, Washington, USA
(7) Department of atmospheric and oceanic sciences, Fudan University, Shanghai, China
(8) Department of Earth system Science, Tsinghua University, Beijing, China
(9) College of Oceanic and Atmospheric Sciences, Ocean University of China, Qingdao, China
(10) Institute of Arctic and Global change, Chinese Academy of Meteorological Sciences, Beijing, China
(11) Institute of Oceanography, Chinese Academy of Science, Qingdao, China

This PDF file includes:

Supplementary Figures 1 to 13

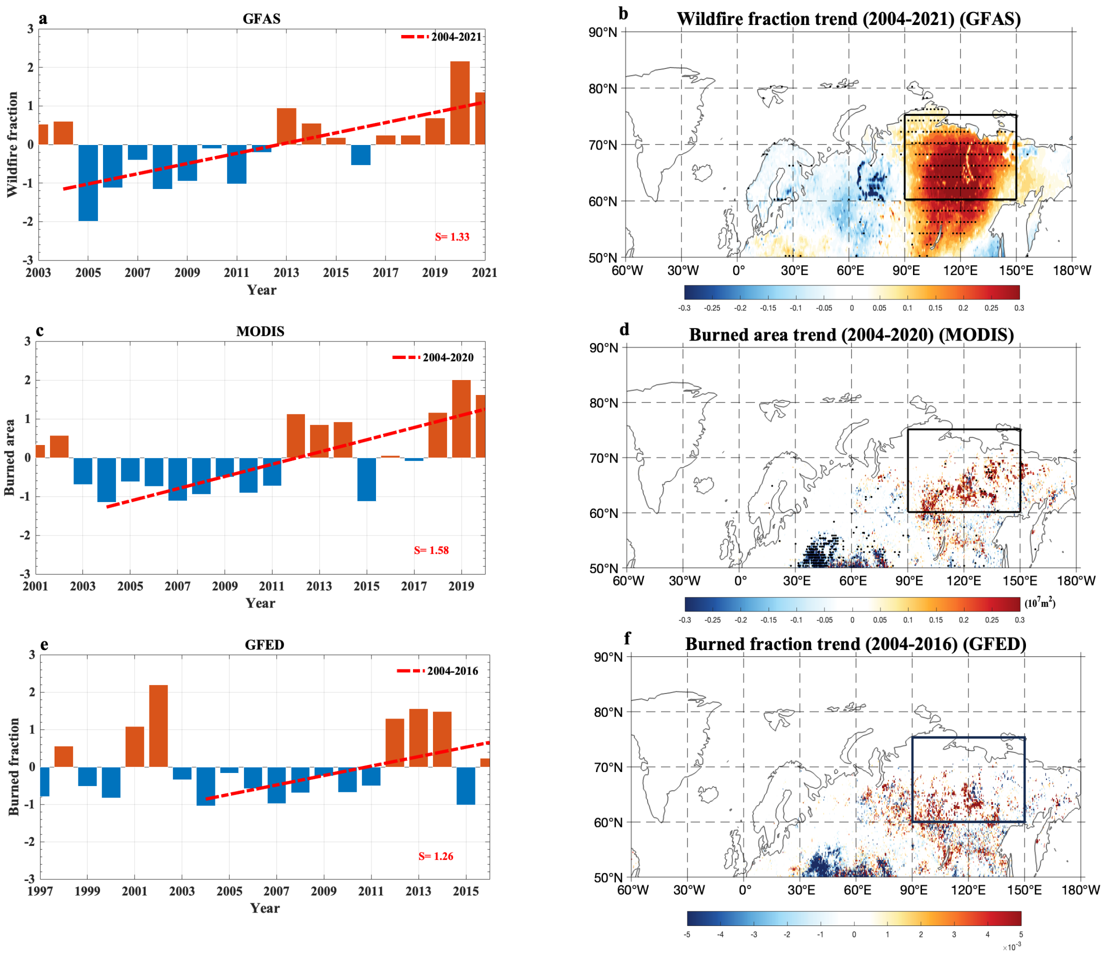

Supplementary Figure 1. Temporal variations of the summer wildfire fraction, burned area and burned fraction and their linear trend patterns for the GFAS, MODIS and GFED data.

(a, c, e) Normalized time series of summer **(**June to August, JJA**)** mean wildfire fraction, burned area and burned fraction anomalies averaged over eastern Siberia (90°-150°E; 60°-75°N) based on **(a)** GFAS during 2003-2021, **(c)** MODIS during 2001-2020 and **(e)** GFED data during 1997-2016. (**b, d, f)** Linear trend patterns of JJA-mean **(b)** wildfire fraction (unit: non-dimensional value per decade) over 2004-2021, **(d)** burned area (unit: m^2 per decade) over 2004-2020 and **(f)** burned fraction (unit: m^2 per decade) over 2004-2016 for **(b)** GFAS, **(d)** MODIS and **(f)** GFED data, where the dot represents the significant region above the 95% confidence level for a two-sided student's t-test.

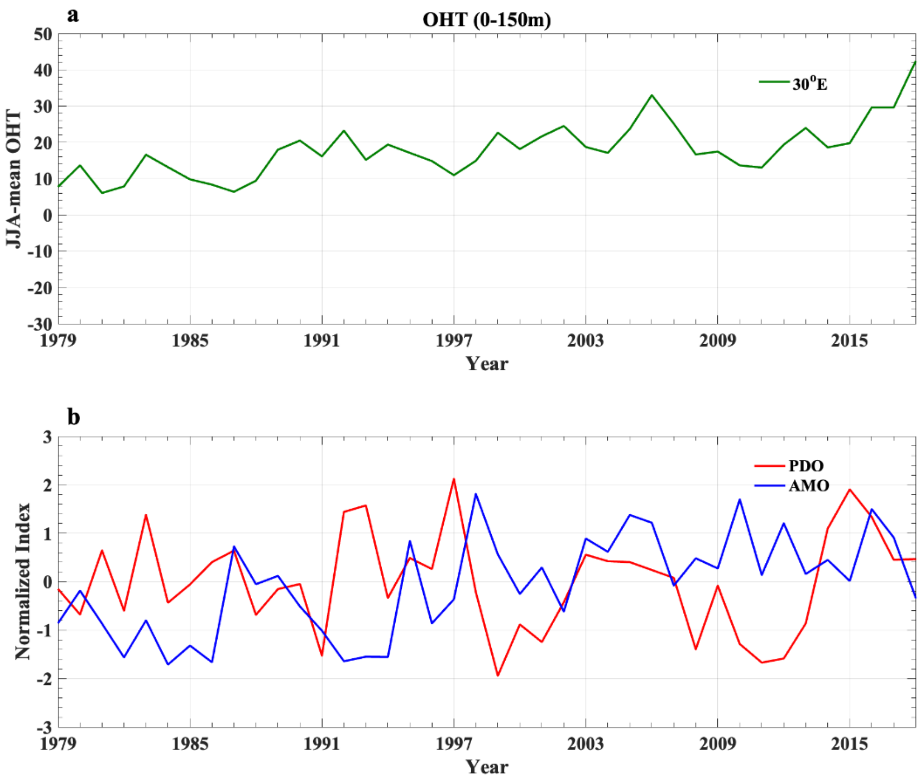

Supplementary Figure 2. Temporal variations of summer (June to August, JJA) mean upper ocean heat transport (OHT) in the Russian Arctic region near the Barents Sea Opening, Atlantic Multidecadal Oscillation (AMO) and Pacific Decadal Oscillation (PDO) indices.

(a-b) Time series of summer (June to August, JJA) mean **(a)** ocean heat transport averaged from the surface to the depth at 150 m (0-150m) and over the region (30°E, 65°-85°N) near the Barents Sea Opening during 1979-2018, and **(b)** Atlantic Multidecadal Oscillation (AMO) (blue line) and Pacific Decadal Oscillation (PDO) (red line) indices.

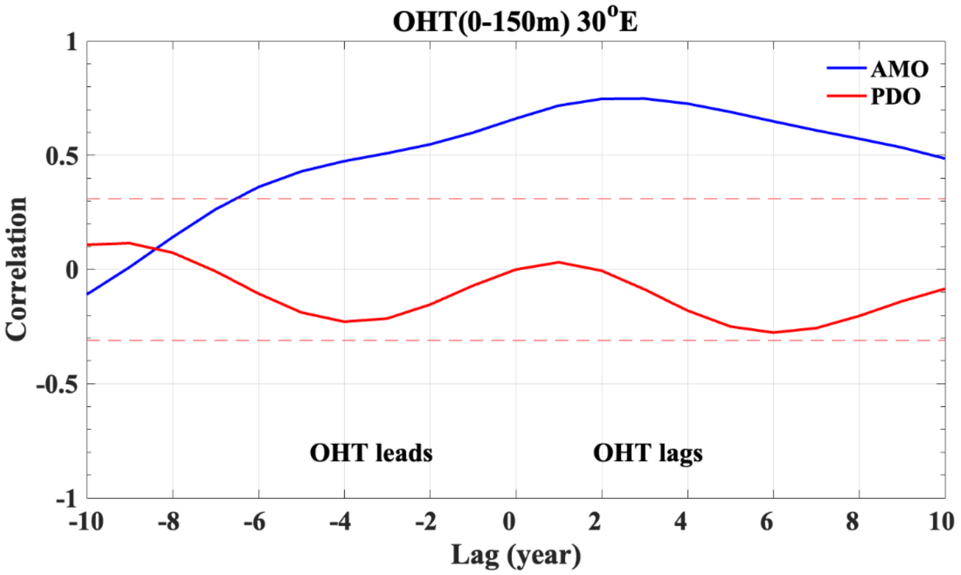

Supplementary Figure 3. Lead-lag correlations of the 9-year low-pass summer Arctic upper ocean heat transport (OHT) with the 9-year low-pass summer Atlantic Multidecadal Oscillation (AMO) and Pacific Decadal Oscillation (PDO) indices.

Lead-lag correlation coefficients of the 9-year low-pass summer (June to August, JJA) mean upper ocean heat transport (OHT) averaged at the location 30°E along the latitude belt (65°-85°N) with the summer Atlantic Multidecadal Oscillation (AMO, blue line) and Pacific Decadal Oscillation (PDO, red line) indices during 1979-2018, where the dashed line represents the 95% confidence level.

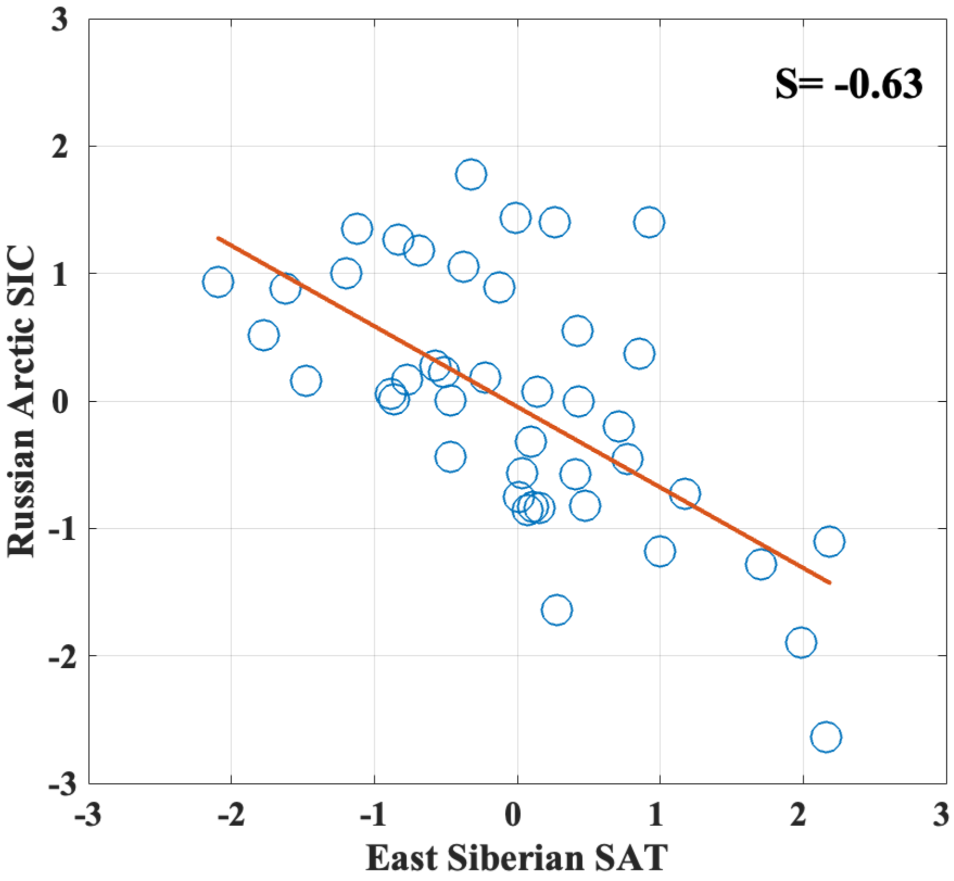

Supplementary Figure 4. Scatter diagram of the summer Arctic surface air temperature (SAT) anomaly over eastern Siberia against the summer Russian Arctic sea-ice concentration (SIC) anomaly.

Scatter diagram of the normalized summer (June to August, JJA) mean surface air temperature (SAT) anomaly averaged over east Siberia (90°-150°E, 60°-75°N) against the normalized Russian Arctic sea-ice concentration (SIC) anomaly averaged over the region (30°-130°E; 65°-85°N) during 1979-2021 for non-detrended data. The slope rate of -0.63 is significant at the 95% confidence level.

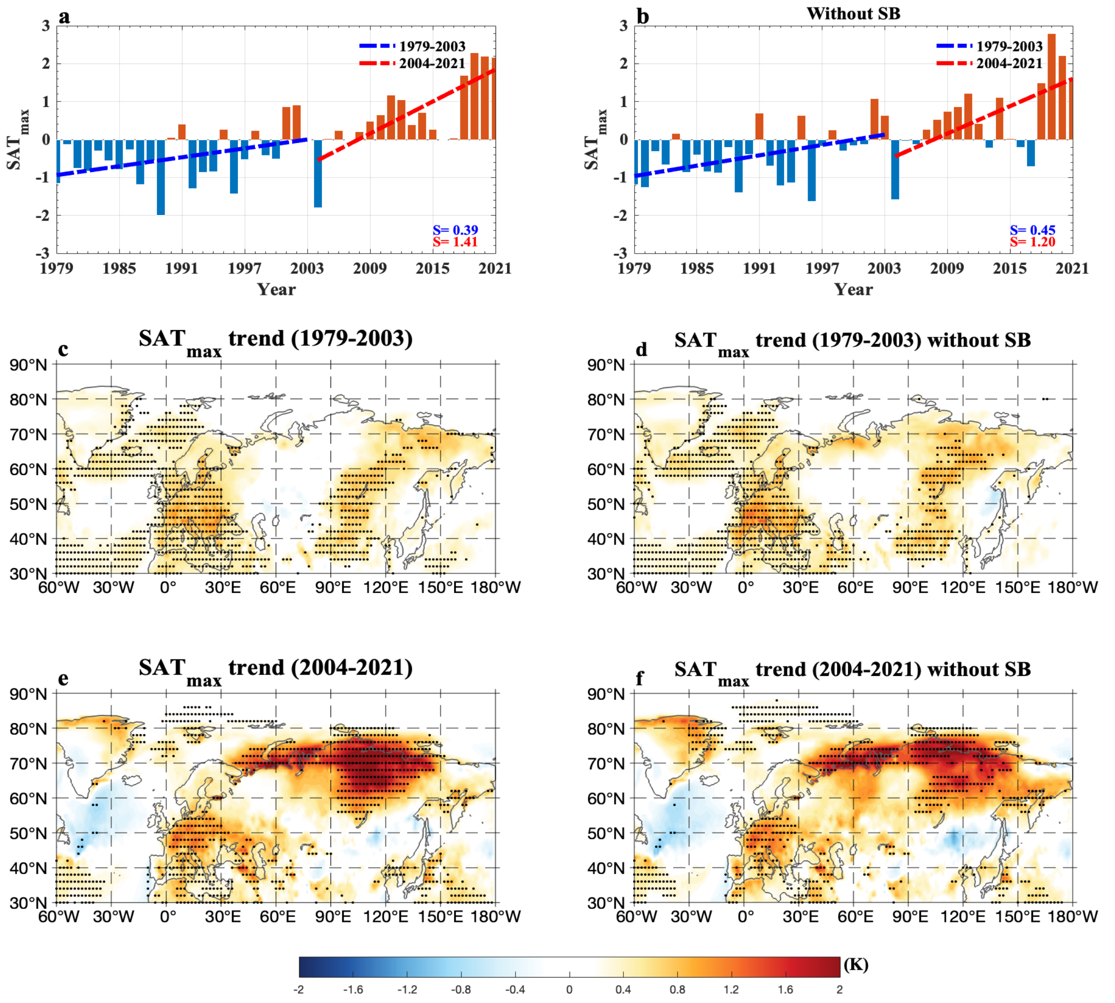

Supplementary Figure 5. Temporal variations of summer-mean daily maximum surface air temperature (SAT_{max}) anomalies averaged over eastern Siberia during 1979-2021 and their linear trend patterns over 1979-2003 and 2004-2021 with and without Siberian blocking (SB) events.

(a-b) Normalized time series of summer (June to August, JJA) mean daily maximum surface air temperature (SAT_{max}) averaged over eastern Siberia (90°-150°E, 60°-75°N) during 1979-2021 based on the ERA5 data, where the dashed blue (red) line represents the linear trends with the slope rates of 0.39 and 0.45 (1.41 and 1.20) standard deviations (STDs) per decade for the cases **(a)** with and **(b)** without Siberian blocking (SB) events over 1979-2003 (2004-2021) (the case without SB events represents that blocking days from lag-10 to 10 days are removed for each SB event). (**c, d, e, f**) Linear trend patterns of JJA-mean daily SAT_{max} (color shading, unit: K per decade) anomalies (**c, e**) with and (**d, f**) without SB events over (**c, d**) 1979-2003 and **(e, f)** 2004-2021, where the dotted regions represent that the trends are statistically significant ($p<0.05$) for the Mann-Kendall test.

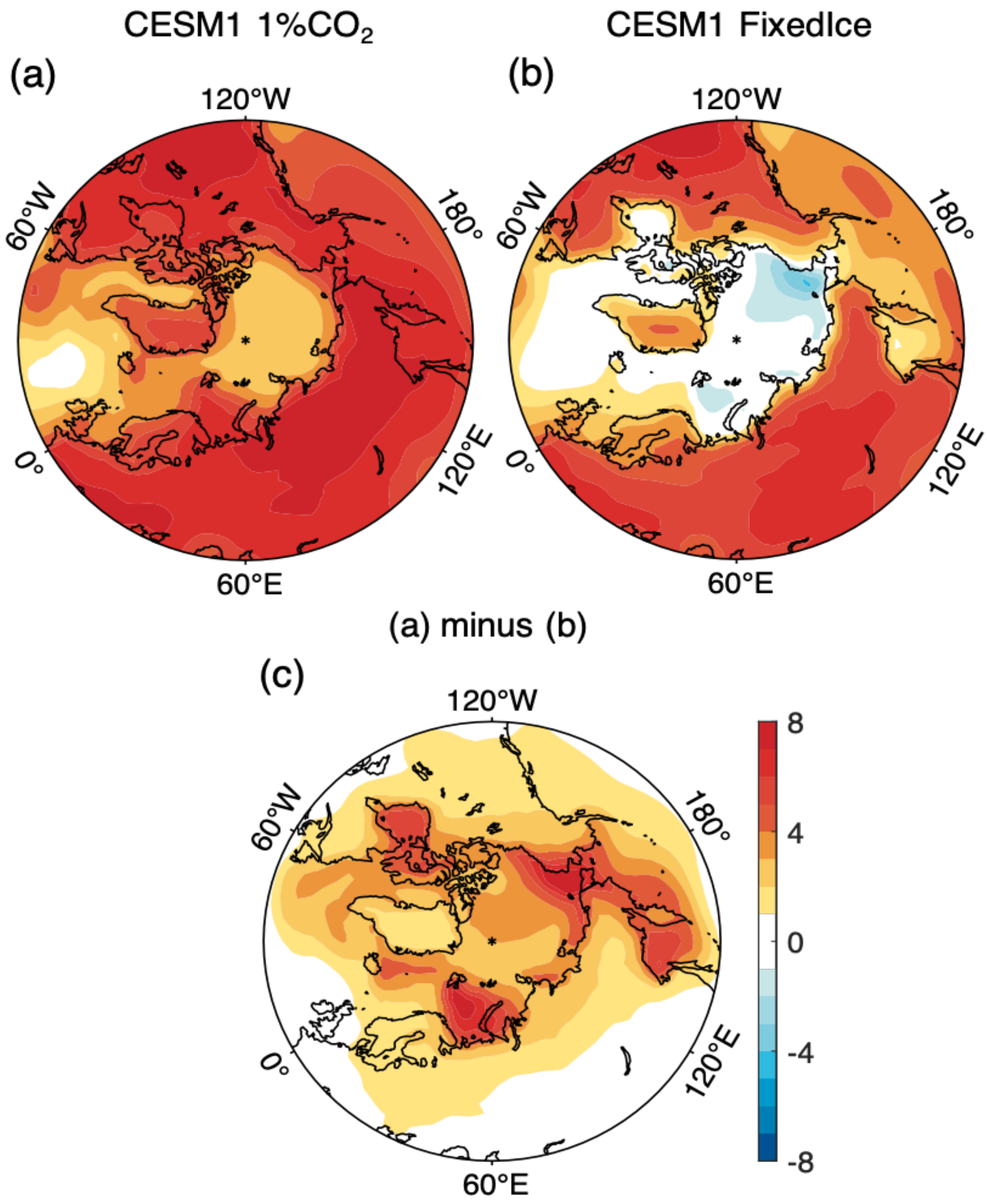

Supplementary Figure 6. Summer mean surface air temperature (SAT) changes north of 45°N for the 1% CO_2 runs without and with fixed sea-ice (FixedIce) and their difference based on the CESM1 model simulations.

(a, b, c) CESM1-simulated summer (June to August, JJA) mean SAT changes (°C, relative to pre-industrial control run) averaged over 45°-90°N and over the 50 years around the time of the second CO2 doubling from 116-165 years for **(a)** the 1% CO_2 run with fully interactive sea-ice (denoted as 1% CO_2 run) and **(b)** the 1% CO_2 run with fixed sea-ice in surface flux calculations (denoted as FixedIce run) and **(c)** their difference, where color shading areas indicate a statistical significance at the 5% level based on a two-sided students t-test.

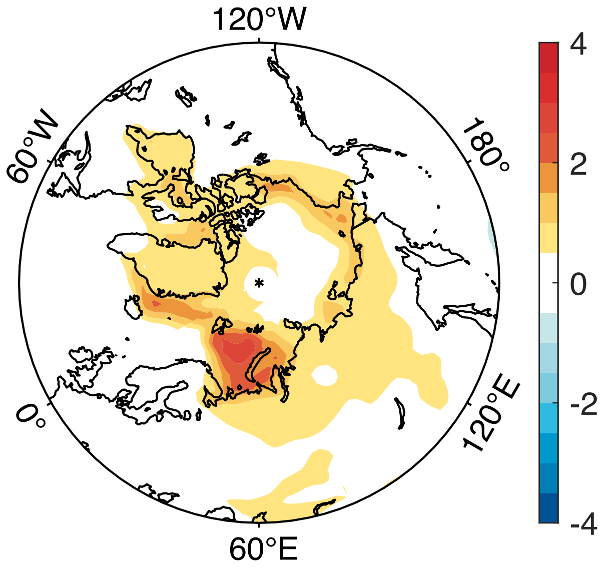

Supplementary Figure 7. Summer mean surface air temperature (SAT) change between future and pre-industrial Arctic sea-ice concentrations.

Summer (June to August, JJA) mean surface air temperature (SAT) difference (ºC) between the atmospheric model simulations with future and pre-industrial Arctic sea-ice concentrations (SIC) but the same sea surface temperature (SST) from the Polar Amplification Model Intercomparison Project (PAMIP) ensemble of CMIP6 models[46]. The PAMIP ensemble consists of 1000 atmospheric model runs from 10 models with specified SIC and SST. The color shading represents the region with the 5% confidence level based on a two-sided students t-test.

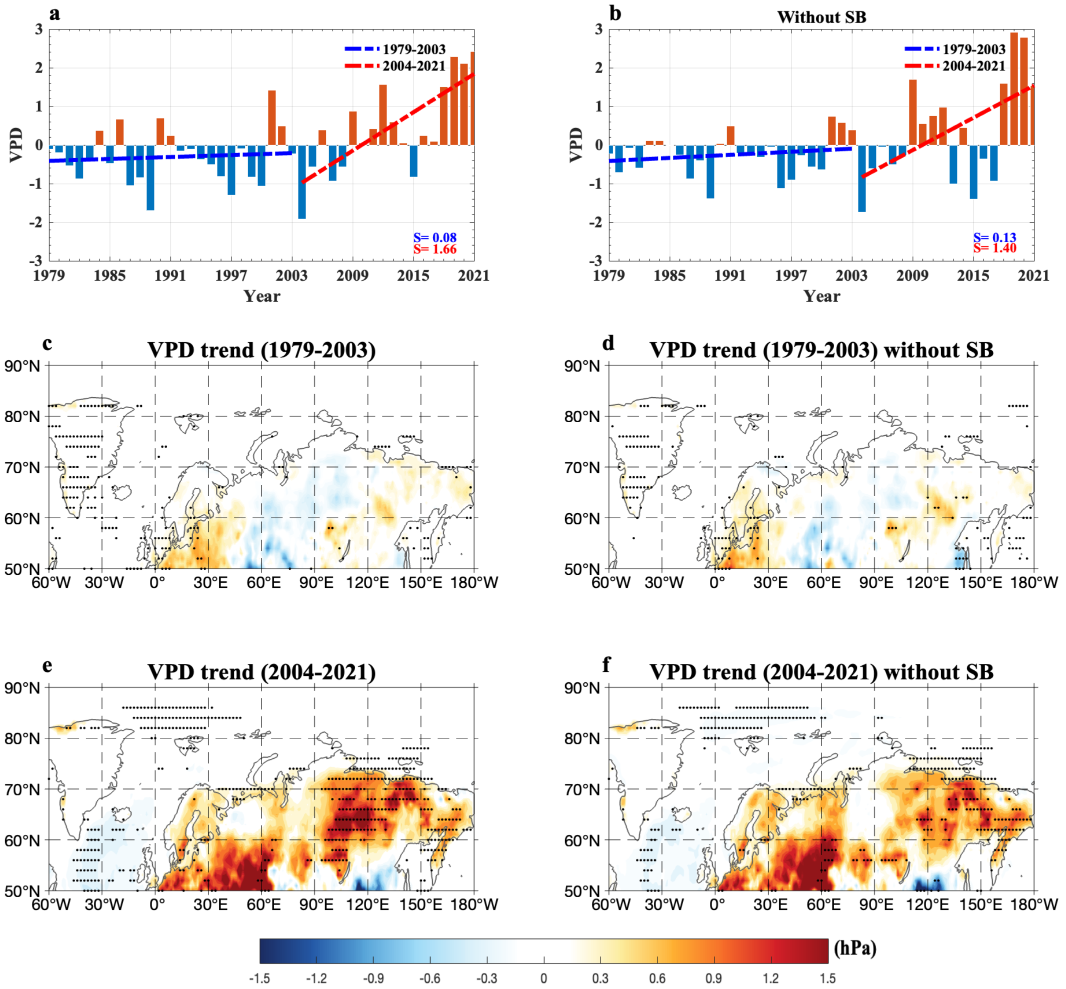

Supplementary Figure 8. Temporal variations of summer mean vapor pressure deficit (VPD) over eastern Siberia during 1979-2021 and its linear trend patterns over 1979-2003 and 2004-2021 with and without Siberian blocking (SB) events based on daily maximum surface air temperature.

(a, b) Normalized time series of summer (June to August, JJA) mean vapor pressure deficit (VPD) averaged over eastern Siberia (90°-150°E, 60°-75°N) during 1979-2021 based on daily maximum surface air temperature (SAT_{max}) from the ERA5 data, where the dashed blue (red) line represents the linear trends with the slopes of 0.08 and 0.13 (1.66 and 1.40) STDs per decade for VPD **(a)** with and **(b)** without Siberian blocking (SB) events over 1979-2003 (2004-2021) (the case without SB events represents that blocking days from lag-10 to 10 days are removed for each SB event). **(c, d, e, f)** Linear trend patterns of JJA-mean VPD (color shading, unit: hPa per decade) over **(c, d)** 1979-2003 and **(e, f)** 2004-2021 for the cases **(c, e)** with and **(d, f)** without SB events, where the dotted region represents the trend area being statistically significant ($p<0.05$) for the Mann-Kendall test.

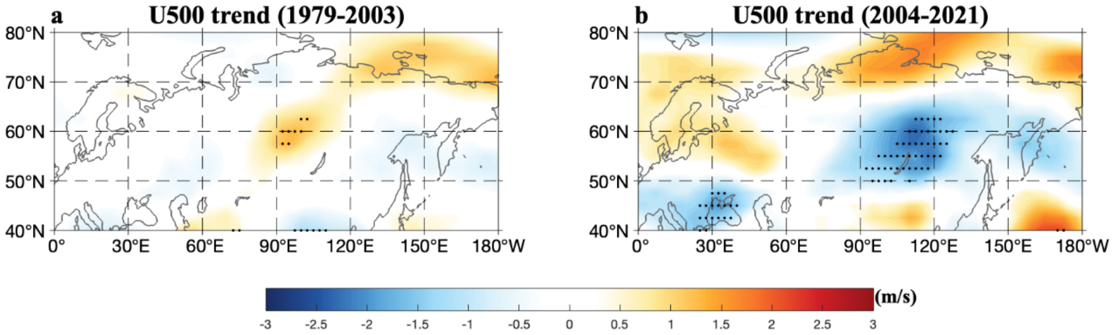

Supplementary Figure 9. Linear trend patterns of summer mean zonal wind anomalies with the effect of Siberian blocking events over 1979-2003 and 2004-2021.

(a, b) Linear trend patterns of summer (June to August, JJA) mean 500-hPa zonal wind (U500) (color shading; unit: m/s per decade) anomalies with Siberian blocking (SB) events over **(a)** 1979-2003 and **(b)** 2004-2021. The dotted regions represent that the linear trends are statistically significant (*p*<0.05) for a two-sided student t-test.

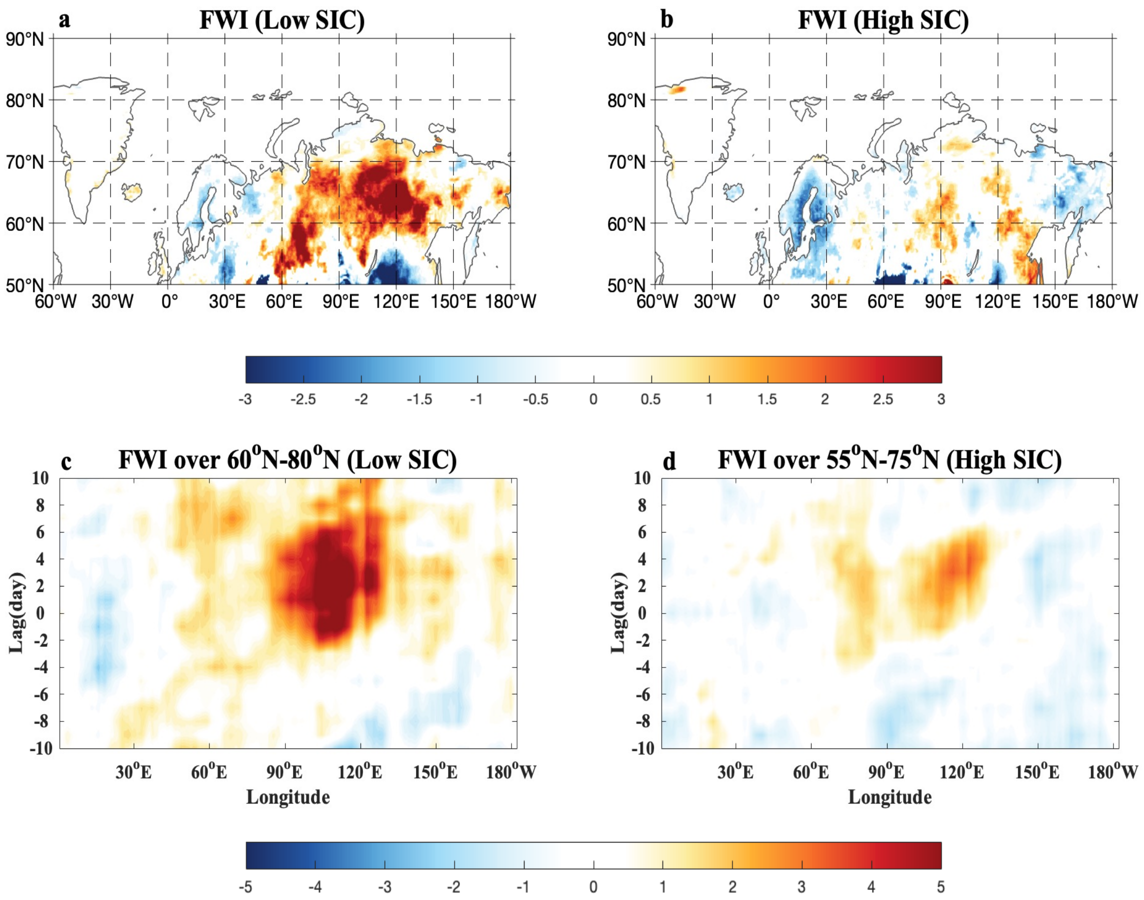

Supplementary Figure 10. Time-mean composite daily fire weather index (FWI) anomaly fields and time-longitude evolution of composite daily FWI anomalies averaged over a latitude belt during the lifecycle of Siberian blocking for different Russian Arctic sea-ice concentration (SIC) conditions.

(a, b) Time-mean composite daily fire weather index (FWI; unit: non-dimensional) anomalies averaged from lag -10 to 10 days of Siberian blocking (SB) events for **(a)** low and **(b)** high summer (June to August, JJA) sea-ice concentration (SIC) conditions during 1979-2021, where lag 0 denotes the peak day of SB. **(c-d)** Time-longitude evolution of composite daily FWI (color shading; unit: non-dimensional) during the life cycle of Siberian blocking events averaged over 60°-80°N and 55°-75°N for **(c)** low and **(d)** high SIC conditions. The red (blue) shading region denotes the positive (negative) FWI anomaly being significant at the 95% confidence level based on a two-sided student t-test.

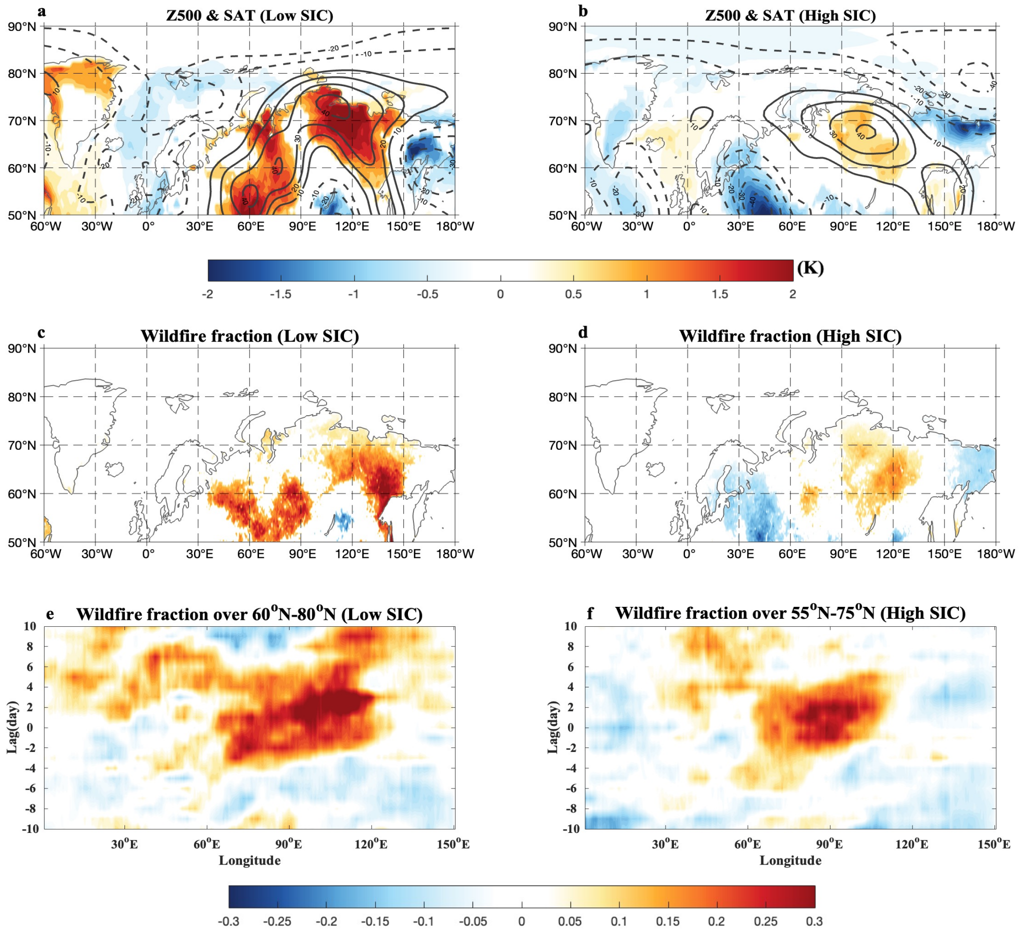

Supplementary Figure 11. Spatial patterns of time-mean composite daily atmospheric fields and wildfire fraction anomalies averaged over the life period of Siberian blocking (SB) during 2003-2021 and time-longitude evolutions of composite daily wildfire fraction anomalies over given latitude regions during the life cycle of SB events under different summer sea-ice concentration (SIC) conditions.

(a-d) Time-mean fields of (**a, b**) composite daily surface air temperature (SAT, color shading, unit: K) and 500-hPa geopotential height (Z500, CI=10gpm) anomalies based on the ERA5 data and (**c, d**) wildfire fraction (color shading, unit: non-dimensional) anomalies based on the GFAS data averaged from lag-10 to 10 days of Siberia blocking (SB) events for **(a, c)** low and **(b, d)** high summer (June to August, JJA) sea-ice concentration (SIC) conditions during 2003-2021, where lag 0 denotes the peak day of the SB. **(e-f)** Time-longitude evolutions of composite daily wildfire fraction (color shading, unit: non-dimensional) anomalies averaged over 60°N-80°N and 55°N-75°N for **(e)** low and **(f)** high SIC conditions during 2003-2021. The color shading regions are statistically significant above the 95% confidence level based on a two-sided student t-test.

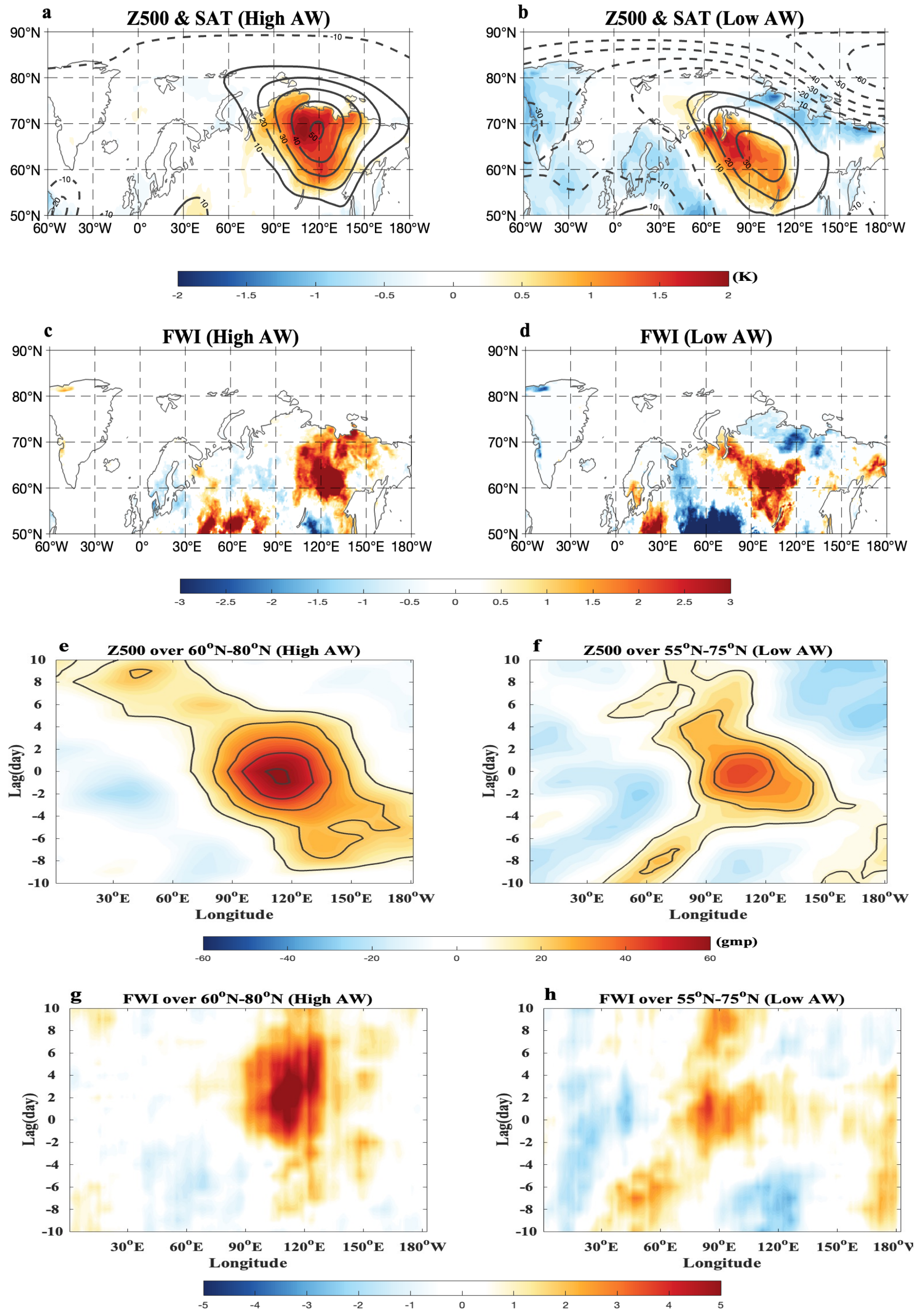

Supplementary Figure 12. Spatial patterns of time-mean composite daily atmospheric fields and fire weather index (FWI) anomalies averaged during the life period of Siberian blocking (SB) events under different Eastern Siberian Arctic warming (AW) conditions during 1979-2021.

(a, b, c, d) Time-mean fields of composite daily (**a, b**) surface air temperature (SAT, color shading, unit: K) and 500-hPa geopotential height (Z500, contour interval=10

gpm) and (**c, d**) fire weather index (FWI, color shading, unit: non-dimensional) anomalies averaged from lag-10 to 10 days of Siberia blocking (SB) events for **(a, c)** high and **(b, d)** low summer eastern Siberian Arctic warming (AW) conditions during 1979-2021, where lag 0 denotes the peak day of the SB. **(e-h)** Time-longitude evolutions of composite daily Z500 (contour interval=10, unit: gmp) and FWI (color shading, unit: non-dimensional) anomalies averaged over 60°N-80°N and 55°N-75°N for **(e, g)** high and **(f, h)** low AW conditions during 1979-2021. The color shading anomalies are statistically significant above the 95% confidence level based on a two-sided Student t-test.

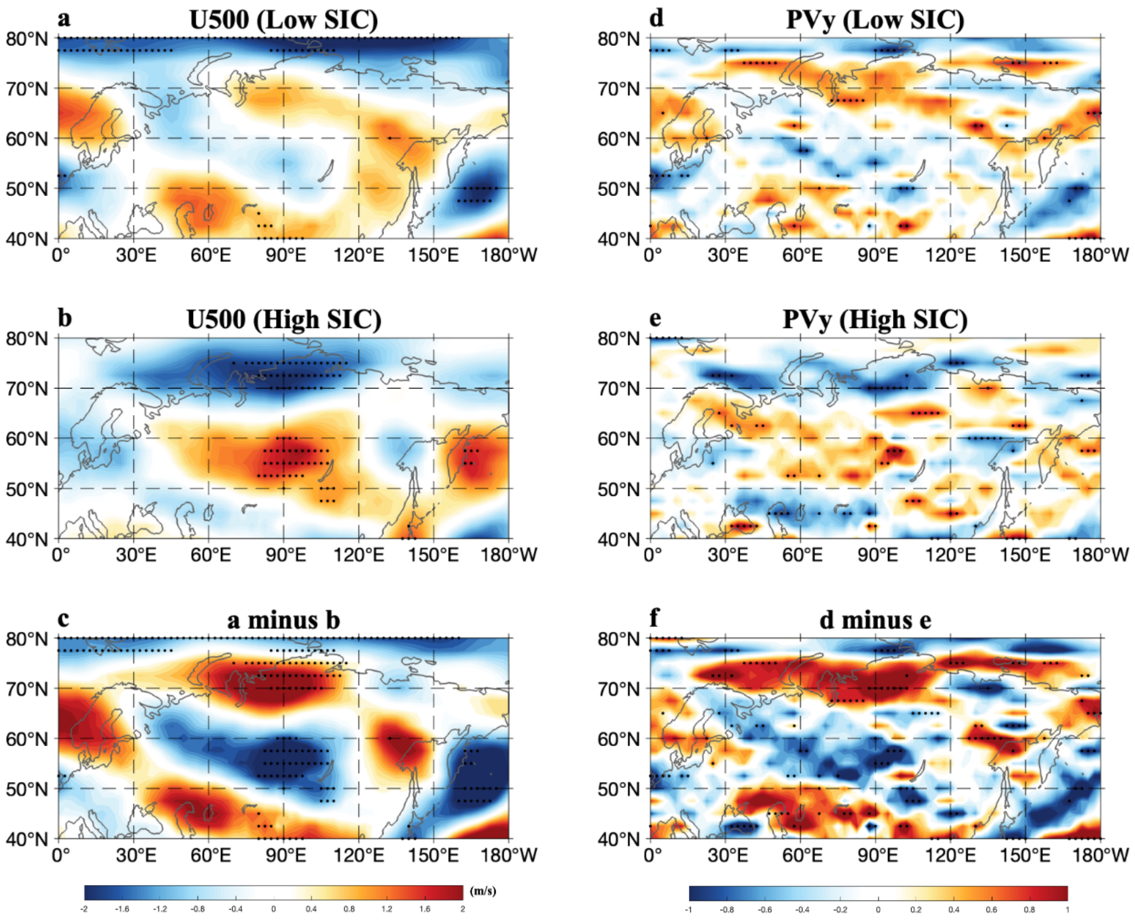

Supplementary Figure 13. The variations of atmospheric background fields with the Russian Arctic sea-ice concentration (SIC).

(**a, b, c, d, e, f**) Summer (June to August, JJA) mean (**a, b**) 500 hPa zonal wind (U500, unit: m/s) and (**d, e**) associated non-dimensional meridional potential vorticity gradient (PV_y, unit: non-dimensional) anomalies without Siberian blocking (SB) events for which the daily fields of the blocking life cycle from lag -10 to 10 days (lag 0 represents the peak day of SB) are removed for (**a, d**) low and (**b, e**) high summer sea-ice concentration (SIC) conditions as well as (**c, f**) low minus high SIC differences during 1979-2021. The dot represents the region being significant at the 95% confidence level for a two-sided student t-test.
